# The Functional Diversity of Nitric Oxide Synthase Isoforms in Human Nose and Paranasal Sinuses: Contrasting Pathophysiological Aspects in Nasal Allergy and Chronic Rhinosinusitis

**DOI:** 10.3390/ijms22147561

**Published:** 2021-07-15

**Authors:** Tomohiro Kawasumi, Sachio Takeno, Chie Ishikawa, Daisuke Takahara, Takayuki Taruya, Kota Takemoto, Takao Hamamoto, Takashi Ishino, Tsutomu Ueda

**Affiliations:** Department of Otorhinolaryngology, Head and Neck Surgery, Graduate School of Biomedical Sciences, Hiroshima University, Kasumi 1-2-3, Minami-ku, Hiroshima 734-8551, Japan; hu0401tk@hiroshima-u.ac.jp (T.K.); chie0324@hiroshima-u.ac.jp (C.I.); pujols@hiroshima-u.ac.jp (D.T.); ttaruya@hiroshima-u.ac.jp (T.T.); kota61@hiroshima-u.ac.jp (K.T.); takao0320@hiroshima-u.ac.jp (T.H.); tishino@hiroshima-u.ac.jp (T.I.); uedatsu@hiroshima-u.ac.jp (T.U.)

**Keywords:** nitric oxide (NO), nitric oxide synthase, isoform, nasal NO, redox pathway, arginase, paranasal sinus, allergic rhinitis, eosinophil, chronic rhinosinusitis

## Abstract

The human paranasal sinuses are the major source of intrinsic nitric oxide (NO) production in the human airway. NO plays several roles in the maintenance of physiological homeostasis and the regulation of airway inflammation through the expression of three NO synthase (NOS) isoforms. Measuring NO levels can contribute to the diagnosis and assessment of allergic rhinitis (AR) and chronic rhinosinusitis (CRS). In symptomatic AR patients, pro-inflammatory cytokines upregulate the expression of inducible NOS (iNOS) in the inferior turbinate. Excessive amounts of NO cause oxidative damage to cellular components, leading to the deposition of cytotoxic substances. CRS phenotype and endotype classifications have provided insights into modern treatment strategies. Analyses of the production of sinus NO and its metabolites revealed pathobiological diversity that can be exploited for useful biomarkers. Measuring nasal NO based on different NOS activities is a potent tool for specific interventions targeting molecular pathways underlying CRS endotype-specific inflammation. We provide a comprehensive review of the functional diversity of NOS isoforms in the human sinonasal system in relation to these two major nasal disorders’ pathologies. The regulatory mechanisms of NOS expression associated with the substrate bioavailability indicate the involvement of both type 1 and type 2 immune responses.

## 1. Background

Nitric oxide (NO), a paramagnetic molecule with an odd number of electrons, is a radical with extreme reactivity that is responsible for many of its biological effects. Transmitted signals mediated by NO are important in the regulation of a variety of physiological and pathological functions, including functions in the nervous, vascular, and respiratory systems [1,2,3,4]. As NO is an uncharged messenger molecule and is highly soluble in hydrophobic environments, it can diffuse freely in cell membranes. In human airways, NO is well known to have physiologically fundamental roles in the homeostasis of both epithelial and endothelial cells, and it stimulates cell proliferation, migration, and differentiation [5,6]. The short half-life and the highly reactive structure of NO require a controlled enzymatic NO synthetic activity that is regulated via complex mechanisms.

Nitric oxide synthase (NOS) catalyzes L-arginine to L-citrulline by the action of the NADPH and tetrahydrobiopterin (BH4)-dependent oxidation, and it produces NO as one of the reaction products [7]. In humans, three NOS isoforms exist: the neuronal (nNOS, NOS1) isoform, the endothelial (eNOS, NOS3) isoform, and the inducible (iNOS, NOS2) isoform [8]. All three isoforms are flavoproteins that contain tetrahydrobiopterin, heme, and an area that is homologous to cytochrome P450 reductase [9,10]. Collectively, eNOS and nNOS are termed constitutive NOS (cNOS). The combination of cNOS and calmodulin (CaM) is dependent on the cellular concentration of Ca^2+^. The rapid Ca^2+^ influx that is induced by various harmful triggers can thus activate cNOS with rapid responses so that cNOS can play a protective role. In contrast, the combination of iNOS and (CaM) does not require Ca^2+^ regulation [10,11,12,13].

In the human nasal cavity, submucosal networks of the blood vessels are constructed mainly by the abundant sinusoid vessels and capillary anastomoses in the inferior turbinate, where NO derived from eNOS diffuses in a gradient manner with nondirectional dispersion. NO performs several regulatory functions in the neurovascular system [14], including roles in smooth muscle relaxation [15], neuronal transmission [4], and the inhibition of platelet aggregation as a part of autonomic nerve function (nasal cycles). NO also has the ability to control the production of surfactants via airway alveolar cells in premature infants [16].

Inhaled NO is universally approved for the treatment of perioperative pulmonary hypertension associated with severe respiratory failure. In this sense, the inhalation of gaseous NO through the nasal cavity is theoretically beneficial [17]. Once formed by eNOS, the vasodilation effects of NO are mediated largely by cyclic guanosine monophosphate (cGMP). In contrast, the expression of iNOS in human airways is rather dependent on transcription factors such as nuclear factor-κB (NF-kB), and is activated by pro-inflammatory cytokines [18,19] including tumor necrosis factor-alpha (TNF-α), interleukin-1beta (IL-1β) [18,19,20,21,22], interleukin (IL)-4, and IL-13 [23,24,25]. Excessive amounts of NO synthesized by iNOS, in combination with other reactive nitrogen oxide species (RNOS), have been considered important mediators of the pathophysiological events underlying a broad spectrum of inflammatory airway responses [26,27].

In the following review, we summarize the current knowledge about NO and NOS and their impact on disease states in common upper airway inflammations, i.e., allergic rhinitis (AR) and chronic rhinosinusitis (CRS). We emphasize the rationale and the potential usefulness of analyses of the fractional exhaled NO (FeNO) and nasal NO, as the information gained by such analyses can be translated into clinical management.

## 2. NOS Activities in the Human Nose and Paranasal Sinuses

### 2.1. NOS Expression and NO Homeostasis in Human Paranasal Sinuses

**Figure 1:**
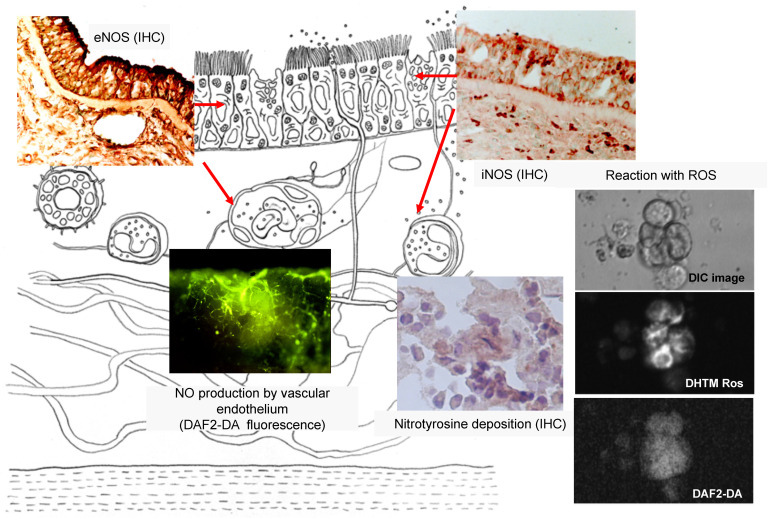
Expression and distribution of different NOS isoforms in human sinonasal mucosa. NOS: nitric oxide synthase; ROS: reactive oxygen species; IHC: immunohistochemistry; DHTM: Ros-dihydrotetramethylrosamine; DAF2-DA: 4,5-diaminofluorescein diacetate.

NO is a free radical that exerts antibacterial effects as a part of the human body’s innate immune defense. As illustrated in Figure 1, epithelial motile cilia covering a large area of the human paranasal sinuses produce bactericidal levels of NO that increase ciliary beating, which is the airway’s major physical defense. NO activates the production of cGMP to activate protein kinase G, which increases ciliary beating and enhances mucociliary clearance [28]. The ciliary beat frequency (CBF) sampled from human sphenoid sinus mucosa increased 24 h after treatment with L-arginine in a dose-dependent manner. A nonspecific NOS inhibitor, i.e., NG-nitro-L-arginine methyl ester (L-NAME), inhibited the L-arginine-induced increase in CBF [29]. The immunoreactivity of both iNOS and eNOS was observed in the ciliated epithelial cells, with eNOS staining being more intense [29]. In a recent study, the stimulation of human nasal epithelial cells with IL-13 under air–liquid interface (ALI) conditions tended to result in an increased level of NO excretion compared to the control conditions [30]. Together, the above-described results demonstrate the potential of the airway epithelial layers to contribute a sizeable counterpart to the excreted NO in the type 2 inflammation that is common to asthma, AR, and eosinophilic chronic rhinosinusitis (ECRS).

Airway pathogens that are responsible for sinonasal infection are susceptible to NO to various degrees [8,31,32]. Interestingly, products with a bitter taste that are secreted from common microorganisms are detected by the receptors of upper-airway epithelial cells. These products elicit T2R (taste family 2 bitter receptor proteins)-activated downstream responses to enhance the production of NO with bactericidal activities [33,34,35]. Bacteriostatic or bactericidal effects of NO may be species-specific. For example, at physiologic concentrations, the common sinonasal organisms *Pseudomonas aeruginosa* and *Candida albicans* are more sensitive to NO, whereas *Klebsiella pneumoniae* and *Staphylococcus epidermis* are more resistant to NO [36].

The production of endogenous NO also exerts antiviral effects against common human respiratory viruses. NO is able to not only inactivate viral particles but also modulate the host immune response that usually triggers an inflammatory response [37]. NO inhibits the activities of viral enzymes (such as proteases, reverse transcriptase, and ribonucleotide reductases) by means of nitrosylation of the amino acids involved in the catalytic process, which leads to interference in viral replication [38,39]. It has also been proposed that the antiviral mechanisms of NO can be applied to the replication of SARS-CoV-2 (severe acute respiratory syndrome coronavirus 2), the virus that causes COVID-19 [40]. NO has the potential to abrogate the replication cycles of SARS-CoV-2 mainly by the S-nitrosylation of specific cysteine residues.

There is TAS2R38 genotype variability in endogenous NO levels in CRS patients with various phenotypes, who generally show lower NO levels in the sinonasal tract [41]. Mucosal exposure to inhaled pathogens also stimulates a defensive swarm of microbiocidal exosomes, which mediates innate immunosurveillance and the defense mechanisms of the human sinonasal epithelium [42]. Lipopolysaccharide (LPS)-stimulated exosomes in mucus sampled from the human nose induced a fourfold increase in NO production by promoting cellular iNOS signaling pathways in in vitro cultures [43].

### 2.2. NOS Expression as an Inflammatory Mediator

Nitric oxide plays both physiological and inflammatory roles, based on the surrounding pro- or anti-inflammatory conditions, as well as the local concentration of NO itself. Free radicals, such as reactive oxygen species (ROS) and reactive nitrogen species (RNS), are cell metabolic products that participate in a variety of cellular events in the airway. These molecules usually have at least one unpaired electron, so they easily react with various substances in the body that are directly exposed to the external atmosphere [44,45]. In the respiratory tract, the function of NO is involved in both the type 1 and type 2 immune responses. In this sense, NO is a key molecule in the Th1/Th2 balance that regulates the evolution of many clinically important diseases. These delicate and complicated effects of NO are dependent on the level and duration of NO production. In general, type 1 inflammation is triggered by low amounts of NO, whereas type 2 cell proliferation is accompanied by the production of IgE and a recruitment of eosinophils that can be induced by higher NO concentrations [23,24,46,47].

Recent advances in the understanding of the important roles of regulatory T cells (Tregs) have revealed a potential new strategy for the control and modulation of mucosal immune responses. NO derived from iNOS and eNOS affects the differentiation of helper T cells and the effector functions of T lymphocytes, and NO is a potential target for therapeutic manipulation [48,49]. The function of T cell-mediated immunity can be regulated by endogenous NO at various concentrations that is generated by iNOS-expressing surrounding cells. Allergic inflammatory diseases are characterized by an increased release of NO and a disruption of Treg cell-mediated tolerance [47,50]. In asthmatic children with AR, the ratio of effector T cells (Th1, Th2, and Th17) to regulatory cells (Treg + Breg) was positively correlated with their FeNO levels but negatively correlated with their forced expiratory volume in 1 s (FEV1) values [51]. In this sense, a functional impairment of Tregs also contributes to the pathogenesis of airway diseases, and NO might have potential roles in the regulation of chronic inflammatory responses through its interaction with Tregs. Future investigations of these topics associated with sinonasal autoimmune and allergic diseases can be expected to yield useful information.

## 3. Monitoring of NO in the Human Sinonasal Pathways

### 3.1. Paranasal Sinuses as a Physiological NO Reservoir

As initially shown by Lundberg et al. [52], the normal human nasal cavity and paranasal sinuses are the major source of NO detected in the respiratory tract. Those authors observed continuous high NO levels up to 3000–25,000 ppb in the maxillary sinuses that contributed to the NO level detected in the nasal airway. Airway ciliated epithelial cells are considered the sites of the highest NOS activity in human airways [6]. Compared to quiet exhalation, a transient acceleration of sinus ventilation produced by humming phonation increased the NO level in the nasal airflow by 15-fold [53]. Although several attempts have been made to measure the production of NO by different paranasal sinuses [54], the relative amount of NO contributed by each paranasal sinus to the human main nasal airways remains unknown.

Interestingly, NOS isoforms detected in the ciliated epithelia in the paranasal sinuses are essentially calcium-independent [55], which is a characteristic that is usually related to iNOS, but the NOS isoforms are constitutively expressed and also resistant to steroid administration. Taken together, these findings suggest that sinus ciliated cells may serve vital physiological functions in nonspecific host defense mechanisms against bacterial or viral infections, and they may help preserve the sterile microenvironment via mucociliary clearance systems. As mentioned above, it is speculated that ciliated cells may have evolved to express various surface receptors that detect bacterial metabolites or foreign materials in order to activate NO-specific defensive pathways [34]. The potent antimicrobial activity of NO may thus be applied in therapeutic modalities.

### 3.2. Nasal NO Measurement

The American Thoracic Society (ATS) has suggested using FeNO to monitor the level of eosinophilic airway inflammation in the lower airways of humans [1,2]. FeNO can also be used as a predictor of responsiveness to an inhaled corticosteroid (ICS) and to evaluate patients’ adherence to anti-inflammatory medications [56,57]. Because of the close linkage between rhinitis, chronic rhinosinusitis, and asthma, the measurement of NO in the nasal cavity provides a promising relevant biomarker of unified airway inflammation [58]. Several methods have been proposed for nasal NO measurement. Two methods are currently recommended in accordance with the ATS/ERS (European Respiratory Society) guidelines: one is nasal aspiration via one nostril with velum closure, and the other is nasal exhalation through a facemask with a fixed flow [1,2]. The latter maneuver is thought to obtain a fraction of endogenous NO with contaminated air passing through the nose with a relatively high flow rate.

The existing data regarding the measurements of nasal NO have provided clear evidence with clinical relevance [59,60]. We examined the local gradients of nasal NO concentrations by using direct online sampling methods, and we compared the levels in different areas inside the nasal cavity [61]. We found that most of the healthy participants showed higher nasal NO levels in the middle meatus (MM) area than in the inferior turbinate (IT) area (mean, 94.8 vs. 48.1 ppb). These results indicate that the maxillary and anterior ethmoid sinuses are the dominant sources of nasal NO detected in the MM area, and they emphasize the role of the paranasal sinuses as a physiological NO reservoir. In this sense, it should be noted that there is a great difference in the background NO output between the upper airways and the lower airways. In the upper airways, there is a higher NO background, and thus an increase in NO (e.g., in allergic rhinitis) tends to be obscured, whereas a decrease in NO (e.g., in primary ciliary dyskinesia or CRS with nasal polyps) is usually more easily detected [54,61,62].

## 4. Allergic Rhinitis

### 4.1. Anatomy and Embryology of the Nose and Paranasal Sinuses

The regions that are affected mainly in AR and those affected mainly in CRS are different in view of the anatomy of the nose and embryological development. Allergic inflammation manifests itself in the inferior turbinate mucosa and is rarely accompanied by nasal polyp formation, whereas the presence of nasal polyps is a hallmark in CRS patients, with polyps mainly in the maxillary and ethmoid sinuses with the compromised ostio-meatal complex (OMC). During embryonic-stage development, the lateral nasal wall is almost completed by 24 weeks’ gestation. By this time, the middle turbinate has developed and ossified from the ethmoid bone, and the inferior turbinate has emerged from different origins, i.e., the maxilla and the lateral cartilaginous capsule. Based on the initial mucosal thickening, turbinate development appears to be a primary process, and meatal ingrowth occurs secondarily [63].

In the nasal airways of healthy humans, NO is produced mainly in the paranasal sinuses; the nasal cavity generates a relatively small amount of NO. The NO levels in the sinus cavity have shown a range of a thousand parts per billion in proportion to the large surface areas. The NO level decreases by approximately one-half in the nasal cavity in a gradient manner [8,64,65]. The role of nasal NO in AR patients has been a matter of debate due to its dual origin, with contributions from both the paranasal sinuses and the turbinate mucosa. The presence of ongoing type 2 inflammation leads to a high production of NO due to the increased expression of iNOS in the inferior turbinate [66,67,68]. This is also supported by a report that the nasal NO levels showed a normal distribution (mean 273.5 ppb) in healthy Chinese subjects without sinonasal diseases and were positively correlated to the subjects’ values of lnFeNO (FeNO log base e) [69].

### 4.2. Increased iNOS Activities in AR

Allergic rhinitis (AR) is characterized by type 2 inflammation that is due to the activation of innate ILC2 cells and acquired T-helper 2 cells, which induces the concomitant release of cytokines including IL-4, -5, and -13 [70]. IgE antibodies are then produced in the nasal mucosa and regional lymphatic tissues in response to the causal antigen’s entry into the mucous membrane [71]. The release of these inflammatory factors can upregulate the iNOS expression in epithelial cells and mucosal inflammatory cells, leading to higher NO generation as clearly documented in a series of human studies [5,6,61,67,68,72,73,74] (Table 1).

In contrast, previous findings have generally shown no significant difference in eNOS gene and protein levels between AR patients and control subjects [66,68]. In accordance with the differential activities of NOS isoforms in the turbinate mucosa, our investigation demonstrated that high nasal NO levels were directly detected on the surface of the inferior turbinate in symptomatic AR patients [68]. The method had the advantage of avoiding the impact of the sizable contribution of NO from the paranasal sinuses [61]. In such an allergic inflammatory microenvironment, pro-inflammatory cytokines and oxidative stress might upregulate the production of iNOS-derived NO through the activation of transcription factors [75]. Excessive amounts of NO can react with ROS to generate peroxynitrite (ONOO−), which can cause oxidative damage to biomacromolecules (lipids, proteins, and DNA), leading to the deposition of cytotoxic substances [61,74,76].

A large amount of a cytotoxic substance such as ONOO− can easily penetrate into cells’ membranes and induce nitrosylation (NT) via tyrosine and cysteine residues. Nitrotyrosine causes oxidative damage to biological macromolecules, especially lipids, proteins, and DNA [77]. We analyzed the concentrations of the inflammatory mediators related to NO metabolism extracted from nasal brushing cells of the inferior turbinate mucosa [61]; elevated levels of NT from oxidized NO metabolites and eosinophilic cationic proteins (ECPs) were concomitantly detected in the inferior turbinate mucosa of allergic patients associated with elevated nasal NO.

The diagnostic values of nasal NO in allergic rhinitis were reported in various patient populations [35,58,60,73,78,79,80]. Because the measurement of nasal NO is noninvasive, easy to perform, and economical, it has become a popular and valuable test for the diagnosis of AR even in children [81]. However, the measurement of nasal NO can be influenced by multiple external factors, including the ambient conditions, the time of day, the subject’s past physical activity, the breathing method, and the analyzer models used [5,82,83]. Further analysis is also required to validate the role of nasal NO and FeNO measurements as objective parameters for the diagnosis of AR independent of other confounding parameters such as nasal airway resistance (NAR). The differences in nasal NO levels among AR patients, asymptomatic atopic subjects, and healthy controls were examined in a large sample of Chinese adults [84], and these levels were observed to be higher in the AR patient group than the other groups. The nasal NO values were related to the FeNO levels, total nasal resistance, and nasal volume within 0–7 cm measured by acoustic rhinometry.

**Table 1:** Studies focusing on the expression and distribution of NOS isoforms and concomitant NO production in allergic rhinitis (AR).

Authors, Year [Ref. No.]	Disease (Sample Area)	Principal Results
Kawamoto et al. 1998 [66]	Perennial AR (IT)	eNOS localization in epithelial and endothelial cells. Increased iNOS staining of epithelial and inflammatory cells in AR patients’ in inferior turbinates.
Kawamoto et al. 1999 [67]	HD mite AR (IT)	iNOS expression of nasal epithelial cells was elevated in the AR group. No difference in iNOS expression after antigen provocation
Takeno et al. 2001 [68]	Perennial AR (IT)	DAF-2 DA imaging showed that epithelial ciliated cells produced a larger amount of NO than nonepithelial inflammatory cells. Preincubation with L-NAME resulted in a 40% decrease in NO production.
Yusel et al. 2008 [73]	Seasonal AR (IT)	iNOS immunoreactivity was higher both in seasonal AR patients and in BA patients. No difference in eNOS immunoreactivity was observed between the groups.
Takeno et al. 2012 [74]	Perennial and seasonal AR, vasomotor rhinitis (IT)	Nasal FeNO levels were higher in perennial AR than in normal subjects or VMR patients, and positive correlations existed between nasal symptom scores and FeNO levels. SAR patients showed increased nasal FeNO levels during the pollen dispersion season.
Takeno et al. 2014 [61]	Perennial AR (IT, MM)	AR patients showed higher nasal FeNO and nasal NO levels in the IT area. No significant difference in the MM area was observed among the groups. AR patients showed higher ECP and NT levels in nasal brushing cells.
Takeno et al. 2017 [78]	Perennial AR (IT)	AR patients showed higher nasal FeNO levels. The optimal cut-off point of the nasal FeNO level was 38.5 ppb for AR diagnosis. No significant correlation was found between nasal FeNO and NAR values.
Hou et al. 2018 [79]	Pollen symptomatic AR (IT)	Increased nasal NO levels were associated with nasal obstruction and NAR. Nasal NO and ECP in secretion were positively correlated in patients with mild-to-moderate nasal obstruction.
Takahara et al. 2019 [65]	Perennial AR (IT, MM)	Nasal NO levels in the IT area in AR patients decreased 2 months after INS treatment. No difference in nasal NO levels in the MM area was observed.

NOS: nitric oxide synthase; AR: allergic rhinitis; BA: bronchial asthma; HD: house dust; IT: inferior turbinate; CBF: ciliary beat frequency; DAF2-DA: 4,5-diaminofluorescein diacetate; L-NAME: NG-nitro-L-arginine methyl ester; FeNO: fractional concentrations of exhaled NO; VMR: vasomotor rhinitis; NT: nitrotyrosine; MM: middle meatus; ECP: eosinophil cationic protein; NAR: nasal airway resistance; INS: intranasal steroid.

Another study evaluated factors that may affect nasal NO in the diagnosis of AR [79]. The study’s findings revealed that the nasal obstruction score, the ECP levels in nasal secretion, and NAR were independently associated with increased nasal NO levels. Our recent investigation also demonstrated that increased levels of nasal NO in AR patients were independent of the nasal airway patency and sensitive enough for a receiver operating characteristic (ROC) curve analysis, with the optimal cut-off point of 38.5 ppb being set to discriminate the AR patients from the healthy subjects [78]. It appears that nasal NO and NAR measurement are two distinctly independent modalities, with the former being more suitable for the diagnosis of AR [85].

The role of allergic inflammation in increased nasal NO levels in AR patients can be examined from another point of view, i.e., comparisons of AR patients with individuals who have vasomotor rhinitis (VMR). We detected no elevation in nasal NO levels in symptomatic VMR patients compared with control subjects, which reflects the fact that different mechanisms underlie the diseases of AR and VMR [74]. Apart from the allergic responses, it is also noteworthy that VMR arises from an imbalance of autonomic input into the nasal mucosa and enhanced parasympathetic responses, which results in increased plasma excretion and glandular secretion [86,87]. Histological damage to the surface epithelium of VMR patients has been reported along with impaired mucociliary clearance without antigen-specific allergic inflammation [88,89]. It therefore appears that different pathological mechanisms of neurogenic inflammation in VMR and IgE-mediated inflammation in AR are likely to be responsible for the different nasal NO levels [90].

### 4.3. Nasal NO as a Therapeutic Parameter

FeNO is a well-established biomarker for type 2 inflammation in bronchial asthma (BA) [1,2], and the FeNO level decreases in response to medical interventions such as treatment with ICS or anti-IL-4/IL-13R antibodies [91,92]. The diagnostic value of nasal NO in patients with BA as a comorbidity has also been investigated in studies based on the “one airway/one disease” theory [93]. The use of FeNO in determining the likelihood of steroid responsiveness is strongly recommended for individuals with BA [3]. Most of the relevant investigations have also demonstrated a reduction in the nasal NO levels of symptomatic AR patients upon treatment with intranasal steroids [65,94,95,96,97,98].

Independent positive associations between perennial allergen sensitization and higher nasal NO levels were observed at both baseline and follow-up periods [92]. In addition, negative associations existed between the daily use of a nasal steroid or ICS and the nasal NO levels during follow-up periods. However, whether nasal NO measurements are useful and reliable enough to monitor the diagnosis and severity of AR and the clinical course remains an open question [35,73,96]. The low reliability of nasal NO measurements is caused by several factors, including the high variability in nasal NO values, anatomical variations in the nose structure, the presence of the nasal cycle, and the comorbidity of sinus disease [97,98].

We recently reported the effects of an INS (fluticasone furoate [FF]) on nasal NO levels in a specific area around the inferior turbinate in untreated AR patients [65]: the nasal NO levels in that area showed a marked reduction after 2 months of ICS treatment, corresponding to an improvement of the patients’ subjective symptoms. However, no significant difference in nasal NO levels was observed in the middle meatus area between the control and AR groups during the study period. These results underscore that the paranasal sinuses are another major contributor to the production of NO in subjects with OMC patency including AR [99].

## 5. Chronic Rhinosinusitis

### 5.1. CRS Phenotypes and NO Production

Chronic rhinosinusitis (CRS) is persistent inflammation of the nasal and sinus mucosa lasting ≥12 weeks, accompanied by two or more nose-related symptoms such as nasal blockage and nasal discharge [100,101]. As with many other chronic diseases, the clinical entity of CRS is considered a disease with heterogeneity. Recent advances in medical devices have enabled the phenotype classification of CRS based on the presence/absence of nasal polyps (NP) revealed by endoscopic imaging (CRSwNP vs. CRSsNP), radiological findings, and the presence of comorbid or systemic illness including BA [100,102,103,104,105,106,107,108].

The establishment of a classification system for CRS endotypes has been attempted, involving histological features such as eosinophilia and specific molecular biomarkers. The combined analysis of the CRS phenotype and endotype could provide insights into treatment responses and pathobiology. Classification based on the level of sinonasal NO production and related enzymes has been a matter of debate, with continuing efforts. The measurement of nasal NO and NOS activities can provide a foundation for new and specific interventions targeting molecular pathways that underlie endotype-specific inflammation in CRS [5,109,110,111].

Patients with CRS usually show pathological features of extensive mucosal dysregulation induced by chronic inflammation. The mucosal damage combined with obstruction of the OMC in these patients contributes to decreased nasal NO levels derived from impaired ciliary activities [104,112]. However, it is not yet known whether the low NO levels detected in CRS are the result of a reduced production of NO by the paranasal sinuses or instead reflect a reduced ability of NO to diffuse in the nasal cavity due to an obstruction of the sinus ostia [8,65].

Most of the existing research has consistently demonstrated a reduction in nasal NO levels in CRS patients, which suggests that nasal NO may be a potential clinical biomarker of sinus inflammation [109,110,113,114,115,116] (Table 2). Negative correlations have been observed between nasal NO levels and the severity of sinus infection, as indicated by computed tomography (CT) scores or nasal polyp scores [59,112,115,116,117]. Ambrosino et al. recently performed a systematic review to investigate the possible link between the nasal NO concentration and CRS phenotypes, and they reported that CRSwNP patients showed significantly lower nasal NO values compared to those of both healthy controls and CRSsNP patients, based on 23 selected articles [118]. Interestingly, the observed difference in nasal NO levels was related to the flow rate of the nasal aspiration, with the difference between cases and control subjects being more prominent when higher aspiration flows were used.

The pathology of nasal polyps is characterized by tissue remodeling, epithelial dysfunction, the activation of innate and adaptive inflammatory responses, and fibrin deposition [21,70,105]. A possible relationship between the formation of nasal polyps and lower NO levels detected in the sinus cavity has been proposed based on the increased amounts of fibrin deposition and prolonged wound healing processes [112]. During wound healing processes of the respiratory mucosa, the deposition of fibrin matrix is replaced by collagen produced by fibroblasts. The biological actions of NO have been demonstrated to be crucial in the wound healing process and tissue regeneration [119,120]. Several lines of evidence indicate that NO induces the expression of collagen in human fibroblasts, and NO-releasing materials are currently being used in a scaffold in wound-repairing beds [121,122]. Decreased NO levels may thus cause a downregulation of tissue collagen production that leads to prolonged wound healing.

Whether decreased levels of nasal NO in CRS are affected by the presence of comorbid AR is another matter of debate [123]. The nasal NO levels in atopic CRS patients were higher than those in nonatopic CRS patients, which indicated that nasal NO could partly reflect the allergic status of the nasal cavity [116]. Another report from the same group further evaluated the impact of sinus inflammation, as detected by CT images on nasal NO levels in AR and non-AR patients; the AR patients without sinus opacity showed higher nasal NO levels (mean, 1180 ppb) compared to the total AR population (mean, 939 ppb), and the non-AR patients with sinus opacity showed the lowest nasal NO levels (mean, 522 ppb) [90]. It is likely that nasal NO could be used to discriminate AR patients who have sinus inflammation with substantial reliability. However, a longitudinal follow-up of a relatively large cohort of BA subjects identified no significant differences in the changes in nasal NO levels in relation to CRS symptoms [92].

### 5.2. NOS Activities in Eosinophilic Chronic Rhinosinusitis

Eosinophilic chronic rhinosinusitis (ECRS) is a refractory and intractable type of CRSwNP. It is histologically defined as >70 residual eosinophils/high-power field (HPF) in nasal polyp tissue [105]. A clinical scoring system named the JESREC (Japanese Epidemiological Survey of Refractory Eosinophilic Chronic Rhinosinusitis) score was established in 2015 for the diagnosis of ECRS based on clinical examination results, i.e., bilateral sinus disease, nasal polyps, CT findings, and peripheral eosinophil counts [124,125]. Several attempts have been made to measure the FeNO or nasal NO levels as a marker for assessing the severity and prognosis factors of ECRS [109,110,112,126]. ECRS patients generally showed higher FeNO levels as a result of a larger proportion of comorbid BA [110,112]. A positive correlation was revealed between FeNO and eosinophilic markers, including blood eosinophil counts, tissue eosinophils in nasal polyps, and JESREC scores [127].

Another study reported that preoperative high levels of FeNO with tissue eosinophilia in nasal polyps (≥70/HPF) were a useful biomarker for predicting the development of asthma symptoms after endoscopic sinus surgery (ESS) [126]. We have demonstrated that, compared to non-ECRS patients, the persistence of eosinophilic inflammation in the ethmoid sinus mucosa of ECRS patients induced a concomitant upregulation of iNOS mRNA, as well as IL-5 mRNA [110]. There was no significant difference in nNOS, eNOS, or TGF-β mRNA levels between the groups. Positive iNOS immunoreactivity was localized mainly in ciliated epithelial cells and associated inflammatory cells with an accompanying deposition of oxidized NO metabolites. Intense nitrotyrosine (NT) staining was colocalized with eosinophil accumulation, and the ECRS patients showed higher rates of NT-positive cells, in line with previous reports [128,129]. We also observed a similar tendency in the local cytokine profiles surrounding the frontal sinus in ECRS patients [111]. The occlusion or stenosis of the frontal ostium with an accumulation of eosinophilic mucin is often inevitable in ECRS patients with compromising clinical problems [130]. It is likely that in ECRS patients, the formation of NT is related to the autotoxic NO mechanism, which is similar to the case with bronchial asthma and substantiates the theory of unified airways.

On the other hand, it remains controversial whether a link exists between nasal NO levels and the severity of ECRS, due (probably) to the multifunctional roles of NO [118]. A recent study reported that nasal NO levels determined by subtracting the nasal FeNO level from the oral FeNO level were significantly lower in the CRS group (both ECRS and non-ECRS) than in the control group, with the ECRS group’s difference being more prominent [112]. The nasal NO levels in the CRS patients were negatively correlated with the degrees of blood and tissue eosinophilia. In a study of a Chinese population, eosinophilic CRSwNP patients also showed lower nasal NO levels than noneosinophilic CRSwNP patients and healthy subjects [123]. In contrast, according to a survey in Denmark, a high level of FeNO was more prevalent in CRSwNP patients compared to controls irrespective of asthma status. Those authors also reported that the nasal NO level was lower in CRSwNP patients compared to controls [131]. These results indicate relative independence between nasal NO and FeNO levels, and they suggest that the physiologically NO-rich nasal airflow is unlikely to affect the orally exhaled air. The results also imply possible mechanisms in which eosinophilic inflammation in ECRS appears either to modulate NOS activities or to inhibit NO diffusion toward the nasal cavity. This theory is also supported by accumulated evidence that type 2-polarized inflammation favors an upregulation of NO production in human airway epithelial cells [23,47].

Although nasal NO has not been established as a useful clinical measure of sinonasal disease [59,132], there is some evidence that the medical or surgical treatment of CRS is associated with changes in nasal NO levels [105,109,133,134]. We prospectively examined the effect of different therapeutic modalities on nasal NO levels in ECRS patients, and we observed that the mean nasal NO levels in the ESS surgical group gradually increased from the baseline (59.3 ppb) to 62.3 ppb at 1 month and to 93.6 ppb at 6 months [109]. Interestingly, the mean oral FeNO levels in this group decreased after surgery, thus suggesting that treatment may result in a recovery of normal NO production by the ciliated sinus epithelium and a cessation of the lower airway inflammation.

One of the nonnegligible problems regarding the use of nasal NO as a potential biomarker for therapeutic assessment is how to identify the source of the nasal NO production responsible for the changes. The treatment of CRS may restore both the iNOS expression of the sinus ciliated cells and the ability of NO to pass through the paranasal sinus ostia. This is particularly important in cases with a unilateral or a limited area of sinus disease. The communicating air flow through the nasopharynx obscures the focused area in the sinonasal tracts. For example, changes in the nasal NO levels of patients with unilateral sinus disease (USD) during the peri-operative period failed to serve as a reliable assessment of disease severity and quality of life (QOL) status [133]. Unlike bilateral CRS patients, the nasal NO levels of the USD patients did not correlate with disease severity. However, the nasal NO levels on both sides were significantly elevated 6 months after ESS in all groups except the fungus group. The patients with fungal sinusitis in that study showed the lowest preoperative nasal NO levels with reliable sensitivity (79.0%) and specificity (87.2%) for a preoperative diagnosis. This is supported by a report that some commensal fungi such as *C. albicans* inhibited iNOS activities in macrophages and blocked NO production in a dose-dependent manner [135]. It is clear that further research is required to elucidate how the post-surgery recovery process of sinus ciliary epithelial cells is functionally related to increased levels of NO production with morphological integrity.

### 5.3. NOS-Arginase Isoform Balance in CRS

The low nasal NO levels that are commonly observed in CRS patients are attributable mainly to the regulatory mechanisms of the expression and activities of the three NOS isoforms. However, there are other rate-limiting factors in cellular NO production, including the availability of intracellular arginine (which is the substrate for NOS) and the activities of arginino-succinate lyase (which converts citrulline back to arginine). This enzyme also plays important roles not only to help synthesize intracellular arginine but also to utilize extracellular arginine for NOS-dependent NO synthesis [7]. In addition, L-arginine is also utilized by arginase (ARG), which is commonly known as the final enzyme in the urea cycle, to form urea and ornithine. Different arginase isoforms, i.e., arginase-1 (ARG1) and arginase-2 (ARG2), have been identified [136]. ARG1 is constitutively expressed (mainly in the liver), whereas ARG2 catalyzes the same reaction but differs in its tissue specificity and subcellular location.

The mechanisms regulating the expressions of ARG1 and ARG2 genes have been proposed to be involved in the induction of airway responsiveness by limiting substrate availability [137,138]. However, controversy remains regarding the clinical significance of arginase activity, which predicts NO deficiency in inflammatory airway diseases. In a study of the relationships among the expressions of NO-related enzymes (i.e., iNOS2 and ARG2), asthma severity, FeNO, and eosinophilic inflammation, the index of iNOS to ARG2 mRNA was reported to be a valuable marker to differentiate severe from milder asthma, despite a tendency for ARG2 mRNA levels to decrease with asthma severity [76].

**Figure 2:**
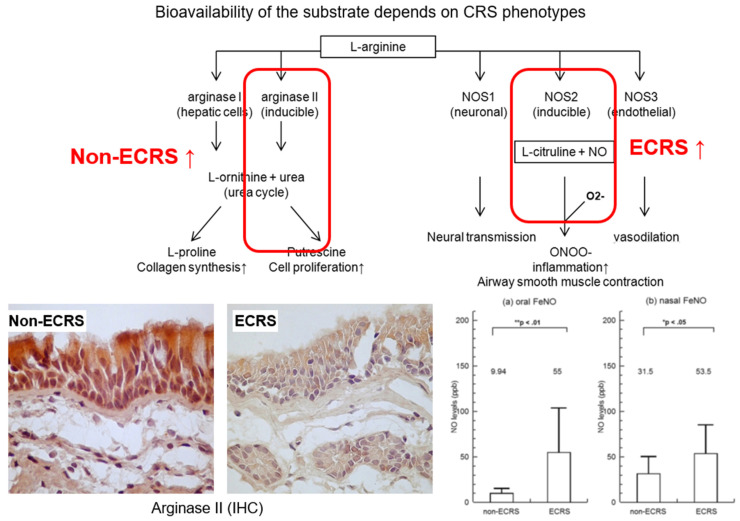
A delicate balance of the NOS/arginase activities in the human sinonasal system modifies the bioavailability of the NOS substrate based on CRS phenotypes. CRS: chronic rhinosinusitis; ECRS: eosinophilic chronic rhinosinusitis; NO: nitric oxide; NOS: nitric oxide synthase; ONOO−: peroxynitrite; O2: superoxide.

In patients with perennial AR, increased expressions of both ARG1 and ARG2 were observed in the inferior turbinate mucosa, suggesting a possible role for the L-arginine–ornithine pathway in the upper airways through competition for the common substrate [139]. One of our studies was the first to shed light on alterations in NO production caused by changes in the NOS-ARG balance in different CRS phenotypes [140]; as illustrated in Figure 2, increased ARG2 activities in CRSsNP patients were associated with significantly lower levels of nasal NO. In contrast, patients with CRSwNP showed significant iNOS mRNA upregulation with concomitant higher levels of FeNO and nasal NO. Our findings and those of other groups suggest that increased ARG2 activity reduces the synthesis of NO in the sinus mucosa of CRSsNP patients and stimulates type 1 (non-eosinophilic) inflammation [141]. The highest level of ARG2 expression was observed in the nonasthmatic and non-AR CRSsNP patients. The pathology of CRSsNP features cellular infiltrations of neutrophils, macrophages, and lymphocytes dominated by type 1 inflammatory cytokines [100,101,105]. It is speculated that moderate expression levels of ARG2 partially inhibit the production of NO so that it can no longer maintain its protective roles but allow an accumulation of NO in a range that is sufficient to promote type 2 inflammatory responses [141].

**Table 2:** Studies focusing on the expression and distribution of NOS isoforms and concomitant NO production in chronic rhinosinusitis (CRS).

Authors, Year [Ref. No.]	Disease (Sample Area)	Principal Results
Chen et al. 2000 [28]	CRS (cultured ethmoid cells)	iNOS expression was augmented by TNF-α and attenuated by dexamethasone, whereas eNOS expression remained unchanged. TNF-α modulated CBF activities through NO production.
Kim et al. 2001 [29]	Normal mucosa (sphenoid sinus)	CBF increased after treatment with L-arginine and was inhibited by L-NAME. Both positive iNOS and eNOS immunostaining were observed in the ciliated cells.
Noda et al. 2012 [109]	ECRS (ethmoid mucosa, NP)	The surgical group showed higher nasal FeNO and lower oral FeNO levels 6 months after ESS, whereas the medical group remained unchanged. Up-regulation and positive immunoreactivity of iNOS was observed in both epithelial cells and submucosal inflammatory cells.
Takeno et al. 2013 [110]	ECRS and non-ECRS (ethmoid mucosa, NP)	ECRS patients showed higher oral FeNO levels and non-ECRS patients showed lower nasal FeNO levels. Positive correlations existed between blood eosinophils and FeNO levels in ECRS patients. Intense NT immunoreactivity was colocalized with eosinophil accumulation and higher NT-positive cells in ECRS patients.
Taruya et al. 2015 [140]	CRSsNP and CRSwNP (ethmoid mucosa, NP)	CRSsNP patients showed increased arginase-2 activities associated with lower nasal FeNO levels. CRSwNP patients showed iNOS upregulation with concomitant higher FeNO levels.
Kubota et al. 2017 [111]	ECRS and non-ECRS (frontal recess mucosa)	ECRS patients showed increased IL-5 and IL-6 mRNA levels in the frontal recess. No difference was observed in TGF-^2^ and iNOS levels.
Yoshida et al. 2019 [112]	ECRS and non-ECRS (NP)	Nasal NO levels were decreased in ECRS patients and negatively correlated with eosinophil levels and CT scores Nasal NO levels remained unchanged after ESS. Reduction in t-PA levels by Th2 cytokines may inhibit iNOS expression.
Vlad et al. 2019 [141]	CRS with BA or AR (Eth)	Arginase 2 expression was higher in CRS patients than controls, especially in nonallergic and nonasthmatic CRSsNP patients. No correlation existed between arginase 2 and IL-13 expression.

AR: allergic rhinitis; BA: bronchial asthma; NOS: nitric oxide synthase; CRS: chronic rhinosinusitis; CRSsNP: CRS without NP; CRSwNP: RS with NP; CBF: ciliary beat frequency; L-NAME: NG-nitro-L-arginine methyl ester; FeNO: fractional concentrations of exhaled NO; ECRS: eosinophilic chronic rhinosinusitis; NP: nasal polyp; ESS: endoscopic sinus surgery; NT: nitrotyrosine; ECP: eosinophil cationic protein; t-PA: tissue-plasminogen activator.

## 6. Conclusions

This comprehensive review highlights that investigations of NO, a gaseous and multifunctional transmitter, remain a source of fruitful research regarding the human nasal system, including the paranasal sinuses. Multiple roles of NO from both physiological and inflammatory aspects are based on surrounding pro- or anti-inflammatory conditions, as well as the local concentrations of NO itself. The diverse backgrounds of the human nasal cavity and paranasal sinuses with anatomical complexity continue to manifest as unelucidated subtleties of the roles of nasal NO. The interpretation of the relationship between upper and lower airway functions based on NO levels is a topic that deserves further attention.

There is consensus as to the diagnostic value of measuring the nasal NO in AR patients, and accordingly, such a measurement has become a popular tool for the objective assessments of the severity of AR and therapeutic efficacy. The classification of CRS phenotypes based on the level of the production of sinus NO and its metabolites has been a matter of debate, and efforts to establish such a classification are ongoing. The measurement of nasal NO and NOS activities can provide a foundation for new and specific biomarkers targeting molecular pathways that underlie endotype-specific inflammation. Focusing on these topics will confirm the significance of the regulatory processes of host signaling pathways for endogenous NO production. Additional or more extended studies can be performed to examine the effects of NOS modulators in treating intractable and refractory diseases in the sinonasal regions.

## Data Availability

Data sharing not applicable. No new data were created or analyzed in this study. Data sharing is not applicable to this article.
